# Optical coherence tomography for ocular surface and corneal diseases: a review

**DOI:** 10.1186/s40662-018-0107-0

**Published:** 2018-06-12

**Authors:** Nandini Venkateswaran, Anat Galor, Jianhua Wang, Carol L. Karp

**Affiliations:** 1 Bascom Palmer Eye Institute, University of Miami Miller School of Medicine, McKnight Building Room 202A, 1638 NW 10th Avenue, 900 NW 17th Street, Miami, FL 33136 USA; 2 Department of Ophthalmology, Miami Veterans Administration Medical Center, 1201 NW 16th Street, Miami, FL 33125 USA

**Keywords:** Anterior segment optical coherence tomography, Ocular surface imaging, Ocular surface lesions

## Abstract

The advent of optical coherence tomography (OCT) imaging has changed the way ophthalmologists image the ocular surface and anterior segment of the eye. Its ability to obtain dynamic, high and ultra-high resolution, cross-sectional images of the ocular surface and anterior segment in a noninvasive and rapid manner allows for ease of use. In this review, we focus on the use of anterior segment OCT, which provides an “optical biopsy” or in vivo imaging of various ocular surface and corneal pathologies, allowing the clinician to diagnose diseases otherwise not visualized by traditional methods. The utility of anterior segment OCT for various anterior segment pathologies is reviewed.

## Background

The rise of new imaging technologies has changed the way ophthalmologists assess the anterior and posterior segment of the eye. These imaging modalities have become instrumental adjuncts to clinical examination for the diagnosis and treatment of several ocular pathologies. There are many imaging modalities that can be employed particularly for the ocular surface and anterior segment including in vivo confocal microscopy, corneal topography, Scheimpflug tomography, high-resolution ultrasound biomicroscopy and optical coherence tomography (OCT) [1].

OCT, which was initially developed for imaging the posterior segment, has shown great promise in systematically imaging the ocular surface and anterior segment from front to back (the tear film, conjunctiva, individual corneal layers, sclera, angle and lenticular structures). Anterior eye segment imaging using 830 nm light wavelength OCT was first demonstrated in 1994 [2]. However, blocked penetration of infrared light by the corneal sclera junction with resultant optical shadowing precluded visualization of trabecular iris angle structures. As such, introduction of transscleral anterior eye segment imaging was achieved by changing the light wavelength from 830 nm to 1310 nm in 2000. In 2005, the first commercially available anterior segment time domain OCT was released [3–6].

**Fig. 1:**
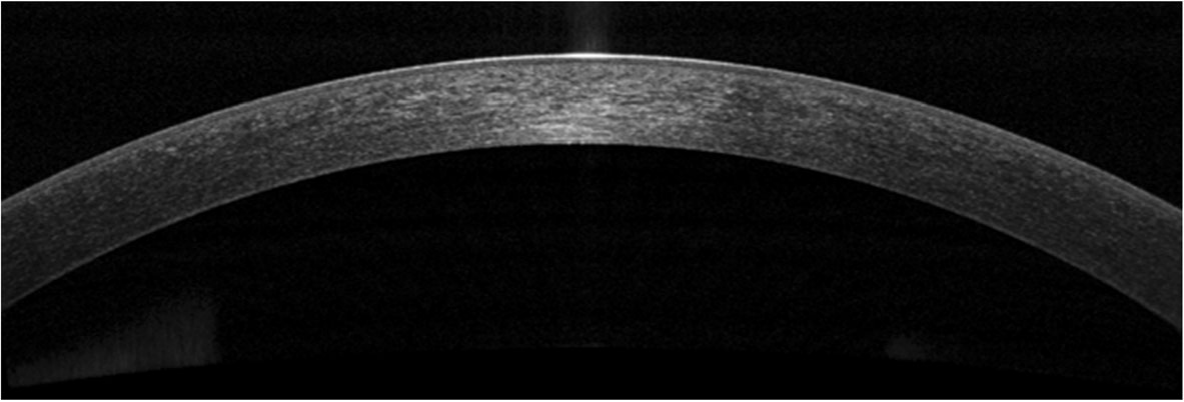
AS-OCT of a normal tear film and cornea. AS-OCT displaying a normal tear film and cornea

However, the transition from time-domain to spectral-domain devices, also known as Fourier-domain OCT, has allowed for faster scan speeds, greater tissue penetrance, and higher axial resolution images due to use of shorter wavelengths of light. Dynamic and rapid acquisition of images can be achieved with axial resolutions ranging from less than 5 μm (considered ultra-high resolution) to greater than 5 μm (considered high resolution). These images provide in vivo, cross-sectional views that elucidate the structural details of various conjunctival and corneal pathologies (Fig. 1) [7]. However, spectral domain OCT devices have the disadvantage of a reduced scan depth compared to time domain OCT machines due to shorter horizontal scan width [3]. More recently, swept-source OCT has emerged as the next advance in OCT technology, enabling simultaneous acquisition of numerous longitudinal and transverse scans to create 3-dimensional corneal, anterior segment and gonioscopic views [8]. There are several high-quality commercially available OCT machines as reviewed in Table 1 [7].

**Table 1:** Summary of characteristics of commercially available AS-OCT machines

Instrument	Company	Measurement type	Approximate axial resolution	Scanning speed per minute
Stratus OCT	Carl Zeiss Meditec	Time-domain	10 μm	400 A scans
Visante OCT	Carl Zeiss Meditec	Time-domain	18 μm	2000 A scans
Slit-lamp OCT	Heidelberg	Time-domain	25 μm	2000 A scans
Spectralis OCT	Heidelberg	Spectral-domain	4–7 μm	40,000 A scans
Cirrus OCT	Carl Zeiss Meditec	Spectral-domain	5 μm	27,000 A scans
OCT SLO	Optos	Spectral-domain	< 6 μm	27,000 A scans
3D OCT 2000	Topcon	Spectral-domain	5–6 μm	50,000 A scans
RT Vue and iVue	Optovue	Spectral-domain	5 μm	26,000 A scans
Avanti	Optovue	Spectral-domain	~ 5 μm	70,000 A scans
SS-1000 CASIA	Tomey	Spectral-domain (swept source)	10 μm	30,000 A scans
Ultra high resolution OCT	Custom build device	Spectral-domain	~ 3 μm	24,000 to 26,000 A scans

In contrast to the currently available devices, our institution has constructed a custom-built ultra-high resolution OCT machine that can acquire images of both high and ultra-high resolution, typically achieving axial image resolutions of 2 to 3 μm. Using a 3-module super-luminescent diode light source with a center wavelength of 840 μm, up to 24,000 A-scans can be generated to produce high-definition cross-sectional images of the area of interest. We routinely use this custom-built OCT at our institution to image both normal and abnormal ocular surface and anterior segment structures for clinical and research purposes [4, 9–13].

Importantly, OCT devices are noncontact and are well-tolerated by patients. The OCT machines can be used by most operators with varying levels of experience and the produced images can be easily interpreted by novice as well as experienced clinicians [14].

In this review, we aim to discuss the various applications of anterior segment OCT (AS-OCT) for dystrophic, degenerative, and neoplastic ocular surface and corneal pathologies as well as provide recommendations for routine use of this beneficial technology in the diagnosis and management of these conditions.

## Applications of anterior segment optical coherence tomography

### Diagnosis and treatment of keratoconus

**Fig. 2:**
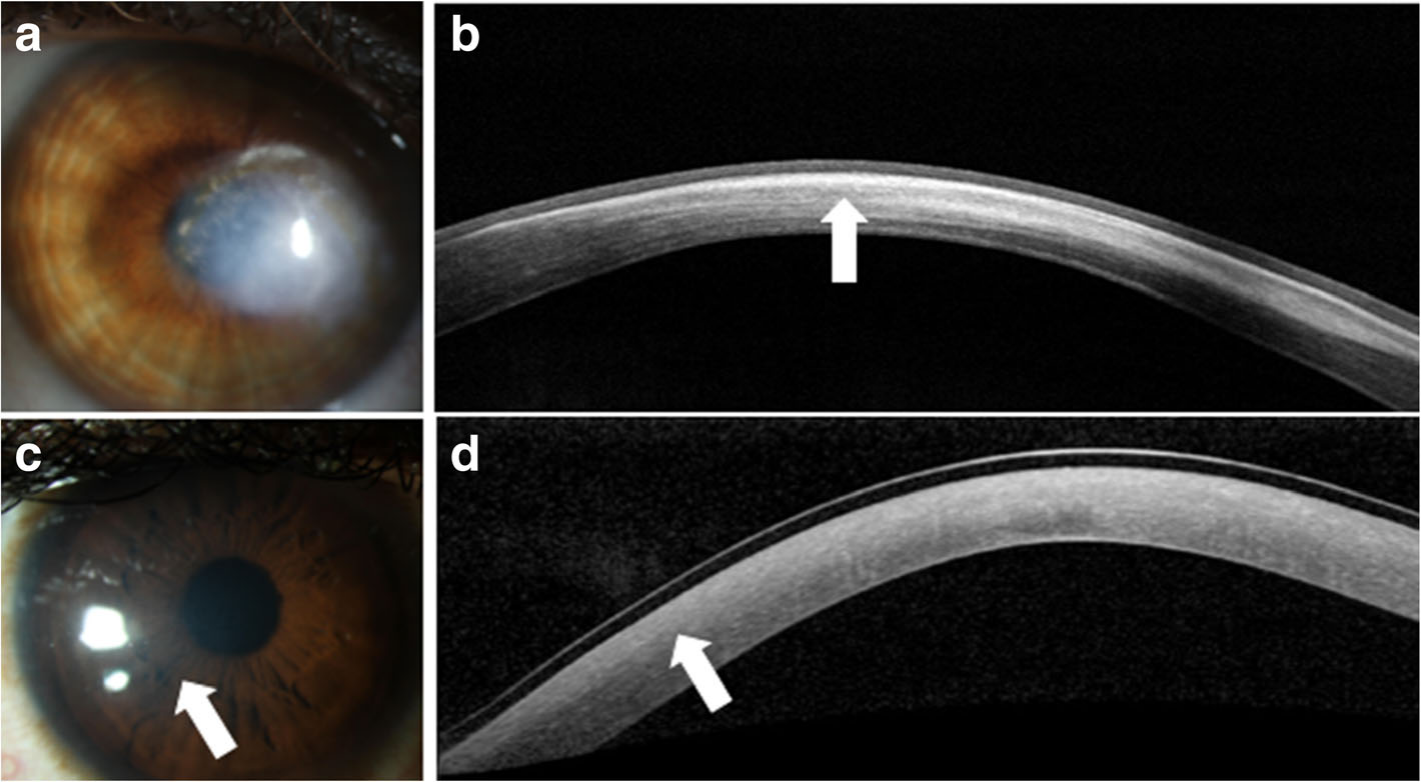
Slit lamp photograph and AS-OCT of keratoconic corneas with corneal scarring. **a** Slit lamp photograph of central scarring in a cornea affected by keratoconus. **b** AS-OCT shows an area of anterior corneal scarring and thinning (arrow). **c** Slit lamp photograph of corneal haze three days after corneal collagen cross linking (arrow). **d** AS-OCT shows a subtle demarcation line in the area of corneal haze (arrow)

Advances in anterior segment imaging have enabled the earlier detection and diagnosis of keratoconus and have allowed clinicians to better characterize the anterior and posterior corneal changes that can occur throughout disease progression (Fig. 2a and b). Imaging modalities commonly used for this condition include Schiempflug tomography, confocal microscopy and OCT [15].

Abou Shousha et al. [10] used our institution’s custom-built ultra-high resolution OCT machine to image and map Bowman’s layer, which is thought to play a crucial role in the pathogenesis of keratoconus. Topographic thickness maps were generated from AS-OCT images to calculate the thickness of Bowman’s layer and specific Bowman’s layer diagnostic indices were proposed. The study found characteristic localized thinning of the inferior cornea in corneas with keratoconus and that the average Bowman’s layer thickness of the inferior cornea was significantly less than the average thickness measured on the superior cornea in corneas with keratoconus. Certain Bowman’s layer indices also showed 100% sensitivity and specificity in the diagnosis of keratoconus and significantly correlated with average keratometry and astigmatic keratometry values [10]. This study showed that ultra high-resolution AS-OCT images can not only help characterize unique Bowman’s layer changes in patients with keratoconus but also provide the means to calculate diagnostic indices that help clinicians more accurately determine this condition.

Importantly, newer commercially available swept source AS-OCT machines are able to scan wider corneal areas and can facilitate creating accurate topographic maps that include measurements from both the central and peripheral cornea and improve diagnostic capabilities [4, 16]. Spectral domain AS-OCT images can be used to characterize the corneal microarchitecture and regional epithelial thickness in patients with early keratoconus and post-operative corneal ectasia. Central epithelial thickness is often significantly thinner in eyes with ectasia and is overall more variable and irregular in ectatic eyes as compared with normal controls, which possibly contribute to changes in corneal topographic values [17]. Early changes in corneal epithelial and pachymetry maps derived from AS-OCT can also help in the early diagnosis of keratoconus in topographically normal eyes as well as in form fruste keratoconus [18, 19]. Additionally, AS-OCT may be used to evaluate epithelial thickness and stromal thinning at the cone and visualize the cornea and anterior chamber in cases of acute hydrops [20].

**Fig. 3:**
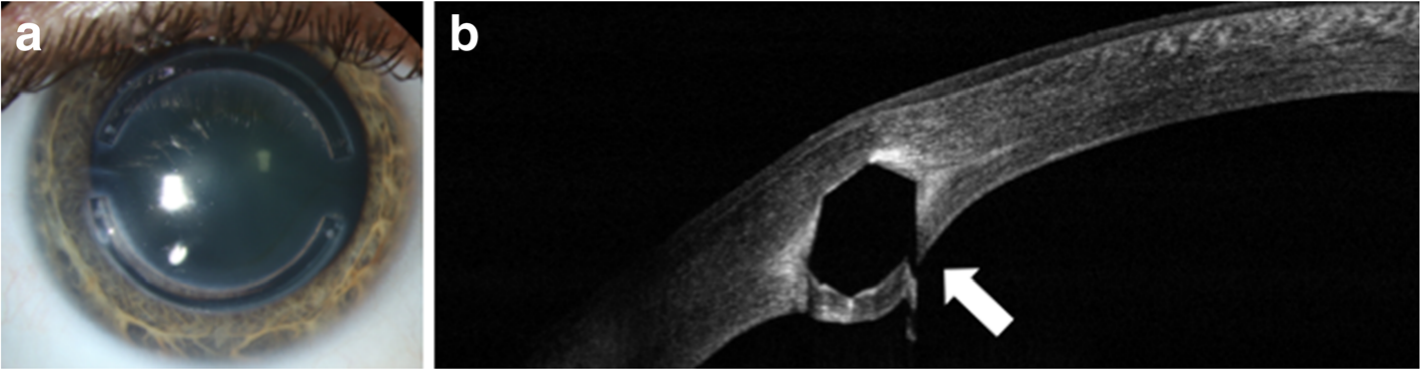
Intrastromal corneal ring segments used in keratoconus. **a** Slit lamp photograph of an intrastromal corneal ring segment used for the treatment of keratoconus. **b** AS-OCT image captures the corneal intrastromal segment and helps assess its location and depth within the cornea (arrow)

AS-OCT is useful in evaluating the effects of treatment for keratoconus, namely cross-linking. Recent papers have proposed the use of AS-OCT to identify corneal demarcation lines (defined by corneal edema and keratocyte apoptosis with changes in stromal reflectivity) to estimate the depth of penetration of different protocols of collagen cross-linking treatments (Fig. 2c and d) [21, 22]. Additional studies are however needed to further evaluate the utility of AS-OCT for gauging cross-linking treatment success. Furthermore, AS-OCT may be implemented to longitudinally evaluate changes in the geometric properties of keratoconic corneas after the insertion of intracorneal ring segments [23] (Fig. 3a) and also assess their position and depth in the cornea (Fig. 3b) [24].

### Ocular surface lesions

**Fig. 4:**
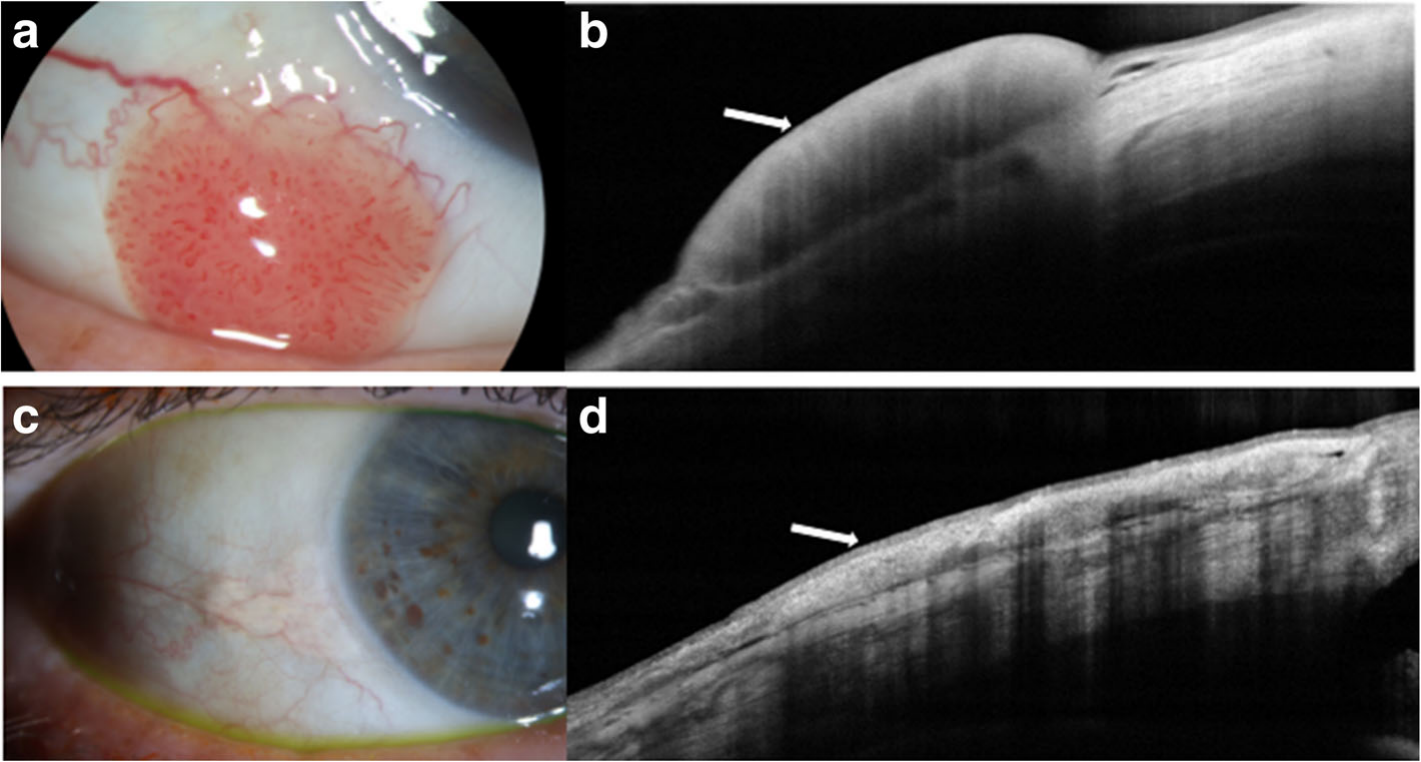
Slit lamp photograph and AS-OCT of ocular surface squamous neoplasia pre and post treatment. **a** Slit lamp photograph of a papillomatous conjunctival lesion. **b** There is an abrupt transition from normal epithelium with thickened hyperreflective epithelium (arrow) on AS-OCT characteristic of ocular surface squamous neoplasia. **c** Slit lamp photograph showing complete resolution of the papillomatous conjunctival lesion after two cycles of 5-fluorouracil. **d** There is normalization of the conjunctival and corneal architecture (arrow) after two cycles of topical 5-fluorouracil on AS-OCT

AS-OCT has shown great promise in the diagnosis and treatment of benign and malignant conjunctival and corneal pathologies. Ocular surface squamous neoplasia (OSSN) is one such pathology that has proven to be uniquely demonstrable on AS-OCT, particularly with devices that can acquire ultra-high resolution images [7]. Clinically, OSSN can present as papillary (Fig. 4a), gelatinous, opalescent or nodular lesions. Definitive diagnosis is traditionally made with incisional and in some cases, excisional biopsies and histopathological analyses. However, with the advent of AS-OCT, distinctive diagnostic features of OSSN have been described that facilitate the diagnosis of OSSN with noninvasive methods. Notably, OSSN is an *epithelial* lesion; distinctive criteria on AS-OCT are a thickened, hyper-reflective epithelial layer with an abrupt transition from normal to abnormal epithelium (Fig. 4b) [7]. In cases of OSSN, these AS-OCT features resolve completely with normalization of the epithelium after successful medical therapy or surgical intervention (Fig. 4c and d) [9]. Moreover, AS-OCT is able to detect subclinical disease that often is not appreciated on slit-lamp examination [4]. As such, AS-OCT serves as a powerful tool for the non-invasive diagnosis of OSSN and can be used to determine the need for treatment initiation as well as monitoring of the disease course.

**Fig. 5:**
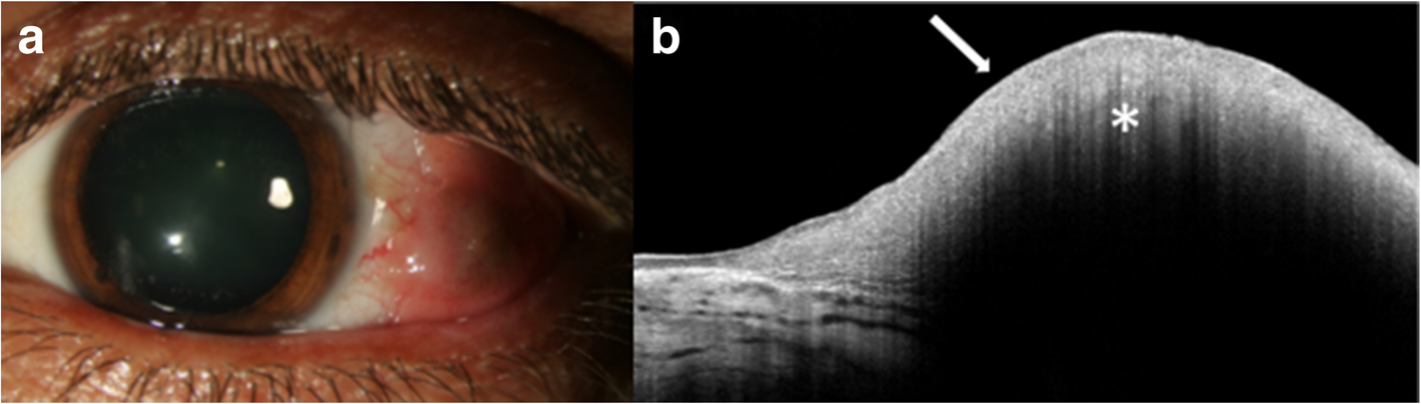
Slit lamp photograph and AS-OCT of conjunctival melanoma. **a** Slit lamp photograph of a mixed amelanotic/pigmented conjunctival melanoma. **b** AS-OCT shows a hyperreflective, subepithelial lesion (asterisk) with thin but hyperreflective epithelium (arrow)

Other lesions that can be characterized by AS-OCT include conjunctival melanomas, lymphomas and amyloidosis [9]. Conjunctival melanomas clinically appear as thickened, raised, pigmented lesions with prominent feeder vessels and surrounding areas of melanosis, but they can also be amelanotic, often making the diagnosis challenging (Fig. 5a) [25]. AS-OCT images show a hyperreflective subepithelial lesion. The epithelium is normal to slightly thick layer of epithelium with variable hyperreflectivity of the basal epithelium (Fig. 5b), which suggests some involvement of the epithelium with atypical melanocytes. This imaging can help to rule in or rule out a pigmented OSSN versus melanoma. When OCT images do definitely rule out OSSN and suggest melanoma, immediate excisional biopsy can be performed. One drawback of these images is that thicker subepithelial lesions can exhibit significant shadowing, which often obscures the posterior limits of or subtle internal details of these subepithelial lesions.

**Fig. 6:**
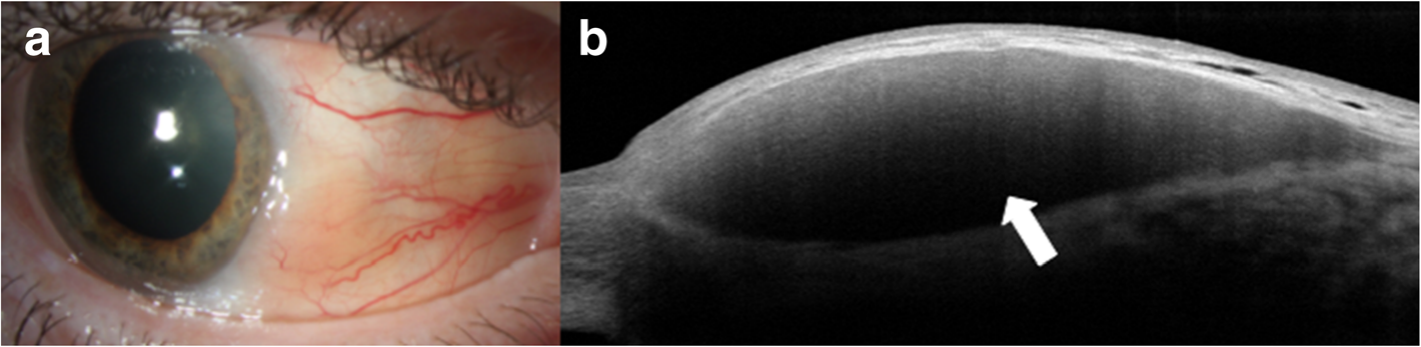
Slit lamp photograph and AS-OCT of conjunctival lymphoma. **a** Slit lamp photograph of conjunctival lymphoma. **b** On AS-OCT, there is a homogeneous, dark, hyporeflective subepithelial lesion with smooth borders and overlying thin epithelium (arrow). The lesion contains monomorphic, stippled, dot-like infiltrates corresponding to the infiltration of monoclonal lymphocytes

Conjunctival lymphomas clinically can present as focal salmon-patch masses, subconjunctival mobile masses or nodules (Fig. 6a) or as chronic follicular conjunctivitis. On AS-OCT, the condition is characterized by a normal layer of epithelium overlying homogeneous, dark, hyporeflective subepithelial lesions with smooth borders. The lesions can often contain monomorphic, stippled, dot-like infiltrates that correspond to the infiltration of monoclonal lymphocytes (Fig. 6b). For both melanomas and lymphomas, AS-OCT images do not always help the clinician obtain a definitive diagnosis as they do for OSSN, but can help guide the differential. Histopathologic analysis of tissue is needed for final confirmation.

**Fig. 7:**
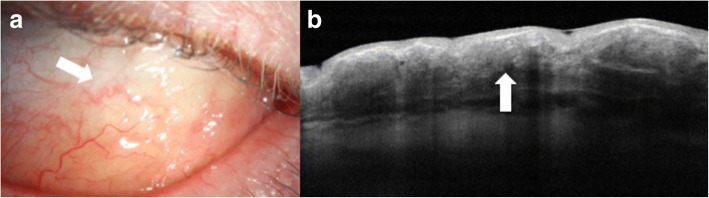
Slit lamp photograph and AS-OCT of conjunctival amyloidosis. **a** Slit lamp photograph of conjunctival amyloidosis (arrow). **b** AS-OCT image of conjunctival amyloidosis showing a heterogeneous, dark subepithelial lesion with irregular borders containing hyper-reflective linear infiltrates that correspond to amyloid deposition (arrow)

Conjunctival amyloidosis can also clinically appear as a yellow or pink lesion on the conjunctiva, similar to lymphoma (Fig. 7a). However, on AS-OCT, images show normal epithelium overlying heterogeneous, dark lesions with irregular borders, as compared with the homogeneous and regular appearance of lymphomas. These *subepithelial* lesions often contain hyperreflective linear infiltrates corresponding with amyloid crystals (Fig. 7b). Once again, histopathology is still the gold standard for diagnosis and is often used for clarification of diagnosis in both primary acquired melanosis and conjunctival amyloidosis [9].

**Fig. 8:**
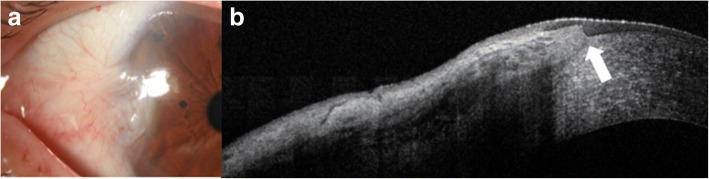
Slit lamp photograph and AS-OCT of pterygium. **a** Slit lamp photograph of a pterygium. **b** AS-OCT image of the pterygium shows a dense, hyper-reflective, fibrillary subepithelial lesion that is between the corneal epithelium and Bowman’s layer (arrow)

When considering benign lesions, AS-OCT can be used to characterize pterygia, conjunctival nevi or primary acquired melanosis. AS-OCT images of pterygia demonstrate a thin or normal layer of epithelium with varying levels of hyperreflectivity overlying a dense, hyperreflective, fibrillary subepithelial lesion that is between the corneal epithelium and Bowman’s layer (Fig. 8a and b). In our experience, AS-OCT has been found to be very sensitive in distinguishing pterygia from OSSN. Several studies have shown that ultra high-resolution AS-OCT can reproducibly differentiate between pterygia and OSSN, namely by statistically significant differences in epithelial thickness and location of the primary lesion (epithelial for OSSN and subepithelial for pterygia) [11, 26].

**Fig. 9:**
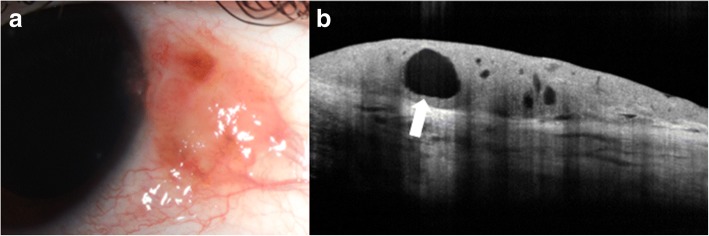
Slit lamp photograph and AS-OCT of a conjunctival nevus. **a** Slit lamp photograph displaying a cystic nevus in a child. **b** On AS-OCT, this lesion is a well-circumscribed subepithelial lesion containing cystic spaces (arrow)

**Fig. 10:**
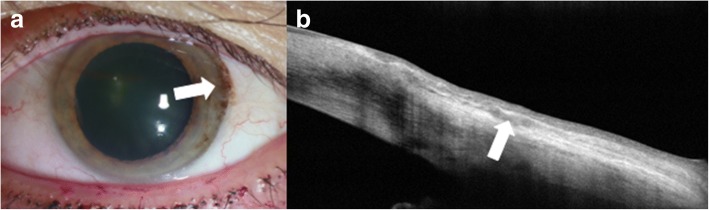
Slit lamp photograph and AS-OCT of primary acquired melanosis. **a** Slit lamp photograph of primary acquired melanosis (arrow). **b** AS-OCT image shows areas of subepithelial reflectivity (arrow)

Nevi, similar to melanomas, often have normal thickness or slightly thickened epithelium overlying a well-circumscribed subepithelial lesion, but unlike melanomas, nevi classically consist of cystic spaces (Fig. 9a), both clinically as well as on AS-OCT (Fig. 9b), which is suggestive of chronicity. Yet, the presence of cysts does not definitively rule out malignancy and a good clinical history and if needed, biopsy, is important to clarify the diagnosis. This technology is especially helpful in the diagnosis of amelanotic nevi often seen in children. In these cases, the cysts may not be clinically apparent, but the AS-OCT can easily allow them to be visualized to assist in the diagnosis. It is important to note that compound nevi can contain a portion of the lesion in the epithelium and substania propria in addition to the subepithelial space. Primary acquired melanosis on AS-OCT images is characterized by normal thickness but moderately hyperreflective basal epithelium with no invasion of the subepithelial space (Fig. 10a and b).

### Corneal pathologies and surgical planning for corneal procedures

AS-OCT can be utilized in the diagnosis and management of dry eye disease [5]. Studies have shown that the tear meniscus can be reduced in different dry eye populations including aqueous tear deficiency or thyroid-associated ophthalmopathy [27, 28]. In patients with dysregulated tear function, lower tear volume can correlate with corneal disease severity. On AS-OCT images, the tear meniscus is precisely measured, and with continuous measurements, the dynamics of the tear meniscus can be trended over time [4, 29, 30].

AS-OCT can be used to image several dystrophic and degenerative conditions of the cornea. With our institution’s custom-built AS-OCT, changes in corneal architecture are captured with two micron axial resolution images. The size, depth, and location of the corneal opacities or deposits can easily be assessed with this imaging modality.

**Fig. 11:**
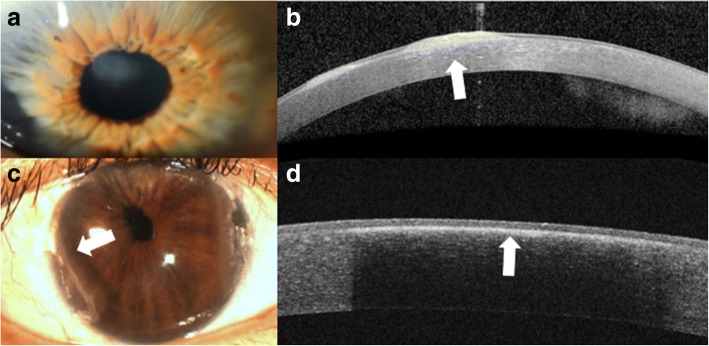
Slit lamp photograph and AS-OCT of a Salzmann’s nodule and band keratopathy. **a** Slit lamp photograph of a central Salzmann’s nodule. **b** On AS-OCT, the nodule is seen as a localized area of hyperreflective material that has replaced the anterior stroma and Bowman’s layer underneath normal epithelium (arrow). **c** Slit lamp photograph of band keratopathy in the peripheral cornea (arrow). **d** AS-OCT imaging shows a thin band of hyperreflectivity along Bowman’s layer with underlying shadowing (arrow)

Salzmann’s nodular degeneration is characterized by localized areas of hyperreflective material that has replaced the anterior stroma and Bowman’s layer underneath the normal epithelium (Fig. 11a and b). This condition can be identified by unique features on slit-lamp examination alone, but when clinical examination is insufficient in distinguishing it from other corneal degenerations, AS-OCT imaging can determine its location and along with diagnostic biopsy, can be extremely useful [12]. Band keratopathy is defined as the deposition of calcium in the Bowman’s layer. On AS-OCT, this can be visualized as hyperreflective material at the level of the Bowman’s layer causing shadowing underneath (Fig. 11c and d).

**Fig. 12:**
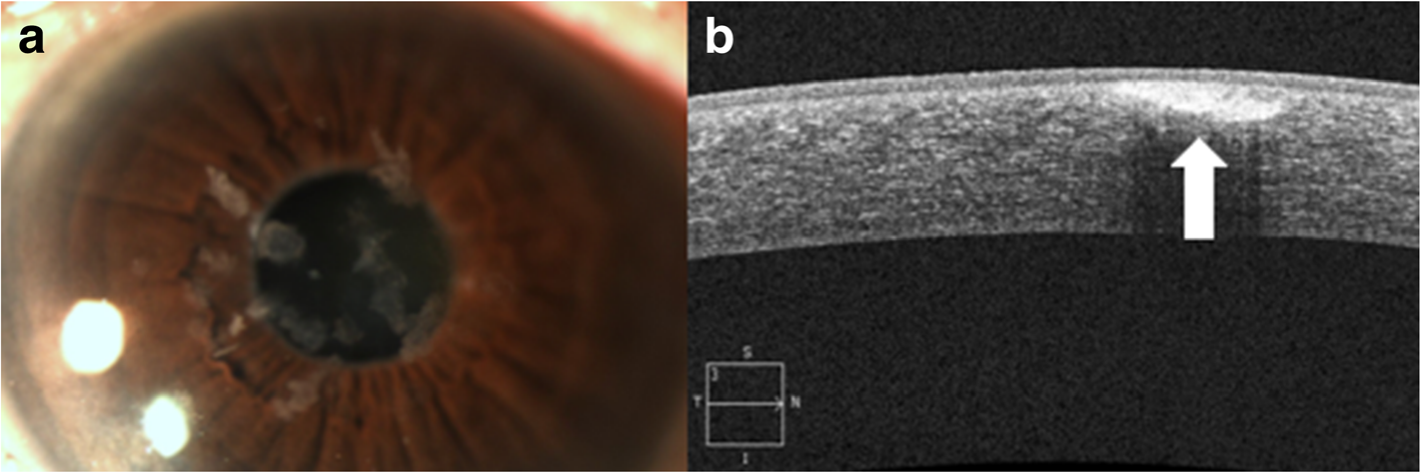
Slit lamp photograph and AS-OCT of granular stromal dystrophy. **a** Slit lamp photograph of granular stromal dystrophy with positive Masson-Trichrome and negative amyloid staining. **b** On AS-OCT, there is hyperreflective material deposited in the anterior stroma with clear intervening spaces (arrow)

Other corneal dystrophies can also be imaged with AS-OCT. When considering epithelial dystrophies, AS-OCT images of anterior basement membrane dystrophy illustrate increased reflectivity of the epithelial basement membranes with areas of basement membrane duplication and intraepithelial hyporeflective cysts. In contrast, Meesmann’s dystrophy is characterized by diffuse hyporeflective microcysts present throughout the epithelium. Dystrophies affecting Bowman’s layer and the anterior stroma can also be imaged. Thiel Behnke dystrophy is characterized by hyperreflective material in a saw tooth configuration deposited on the surface of Bowman’s layer often extending into the epithelium on AS-OCT. AS-OCT images of spheroidal degeneration show cystic structures in Bowman’s layer and in the superficial corneal stroma. Granular dystrophy, which primarily affects the corneal stroma, is often found with hyperreflective material deposited in the anterior stroma with clear intervening spaces (Fig. 12a and b).

**Fig. 13:**
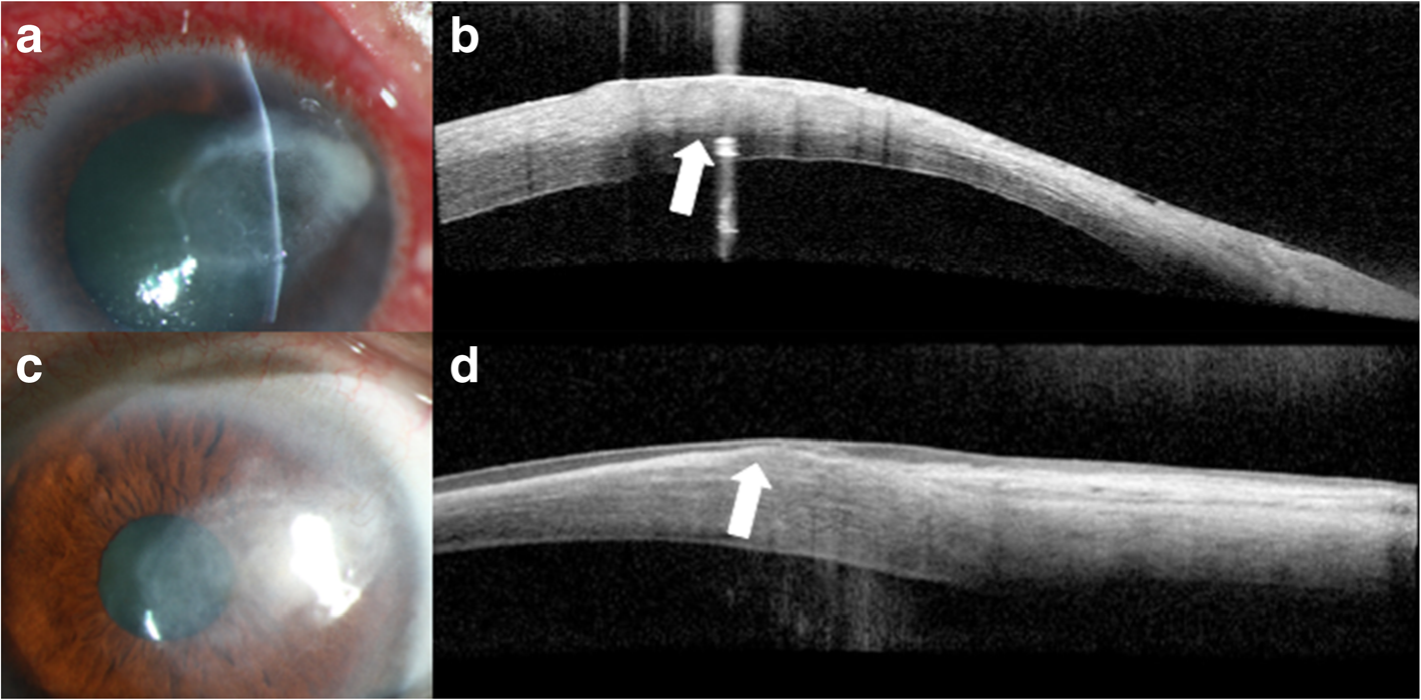
Slit lamp photograph and AS-OCT of infectious keratitis and subsequent corneal scarring. **a** Slit lamp photograph of a patient with contact lens related *Pseudomonas* infectious keratitis. **b** AS-OCT shows diffuse stromal hyperreflectivity and thickening in the area of the infiltrate involving nearly 50% of the stroma (arrow). **c** Slit lamp photograph of a compact, subepithelial scar after infectious keratitis. **d** AS-OCT shows subepithelial thinning and hyperreflectivity in the area of the corneal scar (arrow)

Corneal infiltration in cases of microbial keratitis, which is often seen as hyperreflectivity in the corneal stroma with or without associated retrocorneal membrane formation may be visualized with AS-OCT (Fig. 13a and b). Serial AS-OCT images throughout the disease course can monitor corneal thickness, particularly areas of corneal thinning and scarring, which will appear as areas of subepithelial or stromal hyperreflectivity (Fig. 13c and d) [31]. Particularly in cases of *Acanthamoeba* keratitis, keratoneuritis can identified as highly reflective bands or lines in the anterior to mid stroma on AS-OCT. Sequential images can be used to establish the diagnosis and monitor for resolution [32].

Clinicians identify AS-OCT as useful in elucidating the depth of corneal opacities (i.e. corneal scarring or depositions) or lesions to assist surgeons in determining the optimal surgical procedure for visual rehabilitation [33]. Many times, the extent of corneal opacification can be evaluated by slit-lamp biomicroscopy alone, but AS-OCT can once again be valuable in certain cases where clinical examination proves challenging.

Epithelial debridement or superficial keratectomy can be employed for dystrophies or pathologies limited to the epithelium, subepithelium and/or Bowman’s layer, while phototherapeutic keratectomy can be employed for pathologies limited to Bowman’s layer and/or the anterior stroma. An anterior lamellar keratoplasty can be performed for pathologies extending into the anterior to mid stroma while a deep lamellar keratoplasty can be performed for pathologies extending into the posterior stroma. The AS-OCT can be most helpful in guiding the decision of which procedure to perform. When mostly anterior, femtosecond anterior lamellar keratoplasty is employed [34, 35]. Ultimately, a penetrating keratoplasty can be performed for full thickness or multi-layer corneal pathologies. Endothelial keratoplasty is reserved for pathologies affecting only the corneal endothelium. By understanding the precise location of the corneal pathology with the help of AS-OCT, the clinician can easily use the appropriate surgical intervention to ameliorate visual outcomes.

### Use of AS-OCT for anterior segment surgery

AS-OCT has proven to be an effective tool to monitor the success and complications of several anterior segment surgical procedures including Descemet stripping automated endothelial keratoplasty (DSAEK), Descemet membrane endothelial keratoplasty (DMEK), laser-assisted in situ keratomileusis (LASIK) and even Boston Keratoprosthesis (Kpro) implantation [3].

AS-OCT has also proven to be an excellent intra-operative adjunct for the anterior segment surgeon particularly during lamellar keratoplasty. Intraoperative OCT can be used to assess the effectiveness of Descemet’s membrane stripping and determine the presence of subclinical interface fluid between the host cornea and DSAEK graft that could preclude complete graft attachment [36–38]. Post-operatively, high quality AS-OCT images can allow clinicians to assess graft adherence, graft centration, graft thickness and even epithelial remodeling after DSAEK surgery, all of which can affect the optical quality of corneas post-operatively [5, 39]. Swept-source OCT can even facilitate construction of 3-dimensional corneal topographic maps to quantitate post-operative corneal power, anterior and posterior corneal surface irregularity, intrastromal interface elevation, and pachymetry in post-DSAEK patients [40].

**Fig. 14:**
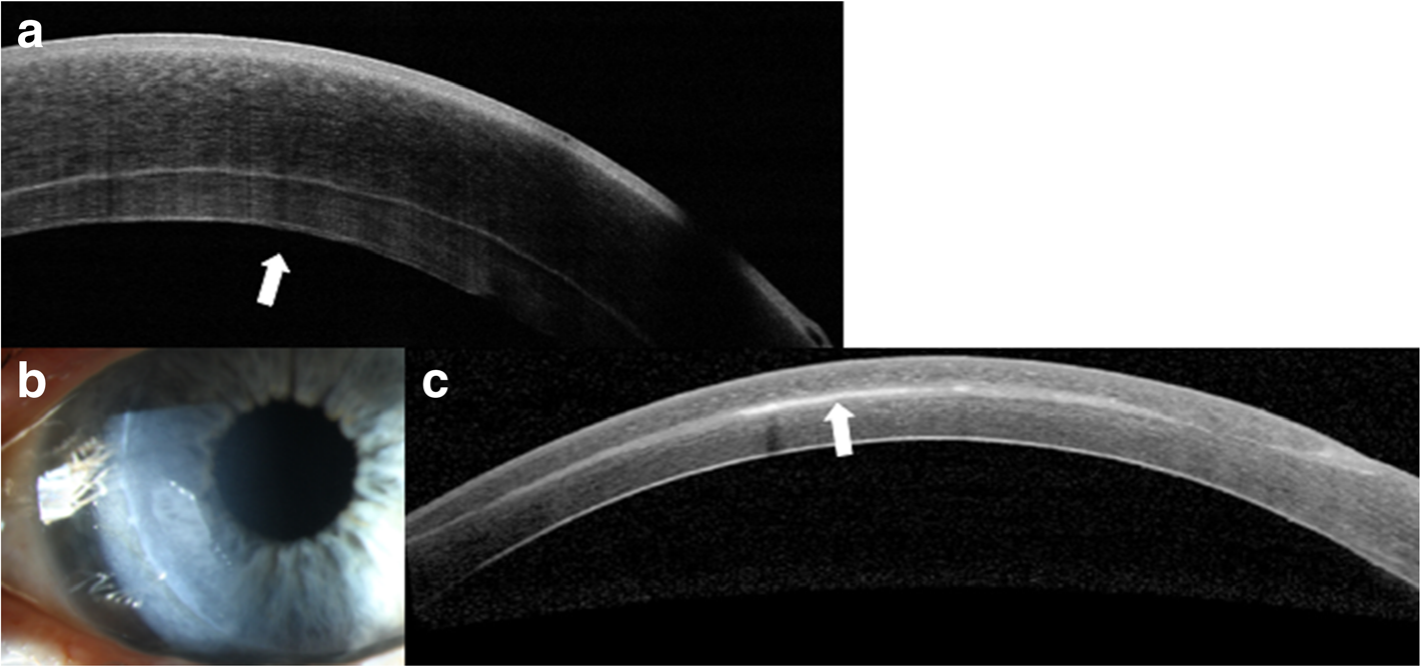
Slit lamp photograph and AS-OCT of an attached DSAEK graft as well as epithelial ingrowth. **a** AS-OCT of an attached DSAEK graft (arrow) post-operatively. **b** Slit lamp photograph of epithelial ingrowth after LASIK. **c** AS-OCT demonstrating the area epithelial ingrowth after LASIK (arrow)

Clinicians can use AS-OCT imaging to help detect early graft detachments that may be challenging to diagnose with slit lamp biomicroscopy or Scheimpflug tomography, particularly in cases using very thin grafts (ultra-thin DSAEK or DMEK) or with persistent post-operative corneal edema or haze. Images evaluating the graft host interface can be obtained intra-operatively with the OCT machine mounted to the operating microscope [36] or post-operatively in the clinic (Fig. 14a). Moutsouris et al. [41] found that in patients with persistent stromal edema after DMEK, AS-OCT added a diagnostic value of 36% in helping discriminate early graft detachments from delayed corneal clearance and was found to be superior to corneal tomography and slit-lamp biomicroscopy in detecting early DMEK graft detachments. Swept-source OCT with protocols capturing limbus-to-limbus and irido-scleral views has also proven effective in detecting early graft detachments after DMEK, particularly when graft detachments were partial and poorly visible due to generalized corneal edema [42].

Implantation of the Type I Boston Kpro can often be associated with complications that can occur secondary to incomplete integration between the Kpro and surrounding cornea. Clinical examination of the Kpro-cornea interface can be difficult, but AS-OCT has shown to be a useful modality to image this interface and facilitate early detection of Kpro-associated complications. Our institution’s ultra-high resolution AS-OCT has been used to capture images of the Kpro-cornea interface with two micron axial resolution [13]. AS-OCT images showed that the corneal epithelium covered the Kpro edge and sealed the potential space at the Kpro-cornea interface in 80% of cases. 20% of cases were found with a gap in the interface that was difficult to detect solely with slit-lamp examination. The authors posed that the lack of epithelial sealing around the Kpro edge might be associated with endophthalmitis. As such, more rapid and accurate identification of incomplete integration of the Kpro-cornea interface with AS-OCT is of great utility and can help clinicians find methods to prevent infection sooner in at-risk patients [13].

AS-OCT may be used to identify flap dislocations after laser-assisted in situ keratomileusis (LASIK). Images can also identify corneal structural changes associated with flap dislocation including macrostriae, flap edema, epithelial hyperplasia and epithelial ingrowth (Fig. 14b and c) associated with LASIK flaps [43].

## Conclusion

With the introduction of high resolution AS-OCT for the ocular surface, cornea and anterior segment, we can ultimately aim to obtain “optical biopsies” of various ocular surface and anterior segment lesions in an era where we are moving towards more rapid and non-invasive diagnostic modalities. This innovative technology helps assess tissue anatomy and evaluate differences in cellular morphology and patterns to distinguish between divergent anterior segment conditions. While there is still room for growth with aspects of this imaging modality, its utility is already quite apparent and it is actively emerging as a promising clinical and research tool.

## Abbreviations

AS-OCT: Anterior segment optical coherence tomography
DMEK: Descemet membrane endothelial keratoplasty
DSAEK: Descemet stripping automated endothelial keratoplasty
Kpro: Keratoprosthesis
LASIK: Laser-assisted in situ keratomileusis
OSSN: Ocular surface squamous neoplasia
